# Integrated analysis of gastric microbiome and clinical features for the diagnosis of gastric neoplasms

**DOI:** 10.1186/s13099-026-00843-3

**Published:** 2026-06-12

**Authors:** Donghoon Kang, Youngjin Shin, Yu Kyung Cho, Jae Myung Park, Seung-Hyun Jung, Myung-Gyu Choi

**Affiliations:** 1 Department of Internal medicine, Seoul St. Mary’s hospital, The Catholic University of Korea, Seoul, 06591 Republic of Korea; 2 Basic Medical Science Facilitation Program, Catholic Medical Center, The Catholic University of Korea, Seoul, 06591 Republic of Korea; 3 Department of Biochemistry, College of Medicine, The Catholic University of Korea, 222 Banpo-daero, Seocho-gu, Seoul, 06591 Republic of Korea; 4 Department of Medical Sciences, Graduate School of The Catholic, University of Korea, Seoul, 06591 Republic of Korea; 5 Catholic Research Institute for Human Genome Polymorphism, College of Medicine, The Catholic University of Korea, Seoul, 06591 Republic of Korea

**Keywords:** Gastric adenoma, Gastric carcinoma, Microbiome, Endoscopic resection, 16S rDNA sequencing

## Abstract

**Background:**

Gastric cancer (GC) progression is closely associated with microbial dysbiosis. However, microbial alterations in the intermediate stage of gastric adenoma (GA) remain under-characterized. This study investigated stage-specific microbiome changes and evaluated predictive models integrating microbial and clinical features.

**Methods:**

We analyzed 231 subjects (129 GA, 73 GC, 29 healthy controls [HC]) using 16 S ribosomal DNA sequencing of paired oral, non-lesional gastric mucosa (GM), and gastric lesion (GL) samples. Random Forest models were constructed using microbial genera and clinical features, validated via leave-one-out cross-validation.

**Results:**

Stepwise microbial dysbiosis was observed during GC progression. Alpha diversity was significantly lower in GC-GL than in HC-GM and GA-GL. *Helicobacter pylori* infection was associated with reduced alpha diversity in GL samples. Genera such as *Streptococcus*, *Neisseria*, and *Haemophilus* were enriched in GC-GL, while *Phocaeicola*, *Faecalibacterium*, and *Bifidobacterium* were depleted. A gastric neoplasm prediction model incorporating 33 microbial genera and six clinical variables (age, sex, BMI, hypertension, smoking, and atrophy) demonstrated superior predictive performance (AUC = 0.830) in distinguishing gastric neoplasms from HCs, compared to models using either microbial genera (AUC = 0.790) or clinical variables (AUC = 0.715) alone. The combined model also accurately distinguished between GA and GC (AUC = 0.807).

**Conclusions:**

The gastric microbiome profile changes dynamically during GC progression, highlighting its potential role in tumor development. Integrating microbial signatures with clinical features enables robust classification of gastric neoplasms and may serve as a valuable adjunctive tool for diagnosis and risk stratification in endoscopic practice.

**Supplementary Information:**

The online version contains supplementary material available at 10.1186/s13099-026-00843-3.

## Introduction

Gastric cancer (GC) remains a leading cause of cancer-related deaths worldwide, with *Helicobacter pylori* (*H. pylori*) infection identified as the primary carcinogen [1]. This infection initiates Correa’s cascade, a stepwise progression from chronic superficial gastritis to atrophy, intestinal metaplasia, dysplasia (gastric adenoma, GA), and ultimately carcinoma [2]. During this process, the loss of acid-secreting parietal cells and subsequent hypochlorhydria facilitate the colonization of non*-H. pylori* bacteria, fundamentally altering the gastric microenvironment [3].

While established risk factors such as advanced age, male sex, family history of GC, high-salt diet, and mucosal atrophy contribute to carcinogenesis [4, 5], *H. pylori* infection alone does not fully explain GC development. Notably, previous studies have demonstrated that as gastric atrophy progresses, the hostile environment leads to the spontaneous loss of *H. pylori*, yet the risk of neoplastic transformation persists [6, 7]. This indicates that additional drivers, particularly the dysbiotic non-*H. pylori* microbiome, may play a pivotal role in the later stages of tumorigenesis.

Recent advances in next-generation sequencing (NGS) have revealed that GC tissues harbor distinct microbial ecologies characterized by reduced diversity compared to healthy gastric mucosa [8–10]. Of note, recent studies have highlighted the pathogenic role of specific bacteria, such as *Streptococcus anginosus*, in promoting gastric inflammation and tumorigenesis [11, 12]. Despite these insights, determining whether these microbial shifts are merely passengers or active drivers in the transition from premalignant lesions to cancer remains a challenge, largely due to the lack of comprehensive profiling in previous cohorts.

Most microbiome studies have focused on comparing GC tissues with adjacent non-lesional mucosa or healthy controls [13, 14], often overlooking the critical intermediate stage of GA. Furthermore, a spatial analysis across the oral cavity, normal gastric mucosa, precancerous lesions, and GC lesions within individual patients is required to elucidate the evolutionary dynamics of the microbiome during the GA-to-GC transition.

Therefore, this study investigated stage-specific microbial signatures by analyzing paired samples from the oral cavity, adjacent normal mucosa, and tumor lesions in patients with GA and GC. Additionally, we aimed to develop and validate an integrated diagnostic prediction model that combines microbial signatures with clinical features to accurately distinguish gastric neoplasms.

## Materials and methods

### Study subjects

This study enrolled a total of 231 subjects, comprising 129 patients with GA, 73 with early GC, and 29 healthy controls (HC). All patients in the GA and GC groups underwent endoscopic resection for their respective lesions. The study protocol adhered to the principles of the Declaration of Helsinki and was approved by the Institutional Review Board of Seoul St. Mary’s Hospital (Approval No. KC19TESI0439). Written informed consent was obtained from all participants. Comprehensive clinicopathological data were collected, including age, sex, body mass index (BMI), comorbidities (diabetes and hypertension), *H. pylori* infection status, pepsinogen I/II ratio, and the final pathological diagnosis of the resected lesions (Table 1).

**Table 1:** Clinicopathologic characteristics of the study population

Characteristic*	Healthy control (*N* = 29)	Gastric adenoma (*N* = 129)	Gastric carcinoma (*N* = 73)	HC vs. Neoplasm (GA, GC) (*n* = 29 vs. 202)		GA vs. GC (*n* = 73 vs. 129)	
				OR (95% CI)	*P*	OR (95% CI)	*P*
Age (mean ± SD)	51.8 ± 14.8	65.0 ± 10.1	68.0 ± 9.3	1.11 (1.07–1.15)	< 0.001	1.03 (1.00–1.07)	0.038
Sex, male (n, %)	13 (44.8)	94 (72.9)	53 (72.6)	3.29 (1.49–7.40)	0.003	0.99 (0.52–1.90)	> 0.900
BMI (kg/m^2^, mean ± SD)	22.9 ± 2.7	25.4 ± 6.4	24.2 ± 2.6	1.20 (1.05–1.38)	0.009	0.94 (0.87–1.01)	0.200
Hypertension, yes (n, %)	6 (20.7)	61 (47.7)	27 (37.0)	3.02 (1.25–8.46)	0.021	0.63 (0.35–1.14)	0.130
Diabetes, yes (n, %)	1 (3.4)	25 (19.4)	12 (16.4)	6.28 (1.27–114)	0.076	0.82 (0.37–1.72)	0.600
Dyslipidemia, yes (n, %)	8 (27.6)	47 (36.4)	23 (31.5)	1.39 (0.61–3.49)	0.500	0.8 (0.43–1.47)	0.500
Smoker, yes (n, %)	5 (17.2)	54 (41.9)	40 (54.8)	4.18 (1.65–12.8)	0.005	1.68 (0.95–3.02)	0.078
Mucosal atrophy							
Closed type (n, %)	26 (89.7)	53 (41.1)	33 (45.2)				
Open type (n ,%)	3 (10.3)	76 (58.9)	40 (54.8)	2.65 (1.86–3.98)	< 0.001	1.02 (0.82–1.28)	0.800
*H. pylori* status							
Not-available (n, %)	17 (58.6)	-	-				
* H. pylori* positive (n, %)	3 (10.3)	53 (41.1)	36 (49.3)	2.36 (0.68–10.9)	0.200	1.40 (0.78–2.49)	0.300
* H. pylori* negative (n, %)	9 (31.0)	76 (58.9)	37 (50.7)				
Location of the lesion							
Non-cardia (n, %)	-	123 (95.3)	69 (94.5)				
Cardia (n, %)	-	6 (4.7)	4 (5.5)				

*The mucosal atrophy classification is based on endoscopic findings, and lesion location applies only to adenoma and carcinoma groups. Unknown *H. pylori* status in the healthy control group represents individuals without available diagnostic information

HC, healthy control; GA, gastric adenoma; GC, gastric cancer; OR, odds ratio; CI, confidence interval; BMI, body mass index

### Sample acquisition

To profile the microbiome across different niches, paired samples were collected from the oral cavity (OC) and stomach. Oral microbiome samples were obtained by swabbing the buccal mucosa after participants had brushed their teeth and rinsed their mouths. Gastric mucosal specimens were collected using biopsy forceps. To minimize potential contamination from the endoscope’s working channel, a sterile plastic tube was first inserted through the channel, and the biopsy forceps were then inserted and retrieved through this tube. Biopsy forceps were disinfected with alcohol after each sampling and before reuse. In patients with gastric neoplasms, tissue samples were obtained from the central lesion (GL) and the adjacent non-lesional gastric mucosa (GM). In the HC group, biopsy samples were obtained from normal GM.

### Sequencing and analysis of bacterial 16 S ribosomal DNA

Samples were stored at − 80 °C until processing. Genomic DNA was extracted using the Maxwell RSC PureFood GMO and Authentication Kit (Promega, Madison, WI, USA). The hypervariable V3–V4 region of the bacterial 16 S rRNA gene was amplified using specific primers, and the amplicons were sequenced on the Illumina MiSeq platform (Illumina, San Diego, CA, USA).

Raw sequencing data were processed using QIIME 2 [15]. Quality filtering and denoising were performed using the DADA2 plugin to infer amplicon sequence variants (ASVs) [16]. Taxonomic classification was assigned to ASVs using the feature-classifier plugin trained on the Greengenes2 V3–V4 custom classifier [17]. Alpha diversity (Shannon index) was calculated based on the genus-level abundance table for each disease group. Beta diversity was assessed using Bray-Curtis dissimilarity and weighted UniFrac distance matrices, visualized via Principal Coordinate Analysis (PCoA) plots. For visualization of group clusters, 95% confidence ellipses were calculated and plotted. Detailed methodologies for alpha- and beta-diversity analyses, differential abundance testing (LEfSe), and functional prediction (PICRUSt2) are provided in the Supplementary Methods.

### Machine-learning approach for the prediction of gastric neoplasms

Predictive models were constructed using the Random Forest algorithm via the randomForest package in R [18]. To capture the core microbial signatures across the entire disease spectrum, feature selection was performed on the entire cohort using linear discriminant analysis (LDA) scores, retaining genera with an LDA score > 3.0 alongside clinical variables. Subsequently, the dataset was randomly split into training and test sets at a ratio of 7:3 for model development and evaluation using the caret package [19]. Samples with missing feature values were excluded from the analysis. To optimize model parameters and prevent overfitting within the training phase, internal validation was performed using leave-one-out cross-validation (LOOCV) exclusively within the training set. The independent test set was reserved strictly for final performance evaluation. Diagnostic performance was evaluated using the area under the curve (AUC) from receiver operating characteristic (ROC) analysis.

### Statistical analysis

Categorical variables were compared using the Chi-squared test or Fisher’s exact test, while continuous variables were compared using the Wilcoxon rank-sum test. To evaluate whether the identified microbial signatures were independently associated with disease status, we performed multivariate linear regression using the log_10_-transformed relative abundance. The model adjusted for potential confounders, including age, sex, BMI, hypertension, smoking, and mucosal atrophy. All statistical tests were two-sided, and a *P*-value < 0.05 was considered statistically significant. All analyses were performed using SPSS software (version 26.0; IBM Corp., Armonk, NY, USA).

## Results

### Clinicopathological characteristics

A total of 231 subjects were included in the analysis: 129 with GA, 73 with GC, and 29 HC. Baseline clinicopathologic features are summarized in Table 1. A significant male predominance was observed in the GA (72.9%) and GC (72.6%) groups compared to the HC group (44.8%). While the distribution of closed- and open-type mucosal atrophy was similar between GA and GC, the prevalence of open-type atrophy was significantly higher in GA (58.9%) and GC (54.8%) than in HC (10.3%). *H. pylori* infection rates were 41.1% in GA and 49.3% in GC patients. Most neoplastic lesions were located in the non-cardia region. Detailed characteristics are summarized in Supplementary Table S1.

Univariate analysis identified advanced age (*P* < 0.001; odds ratio [OR] 1.11, 95% confidence interval [CI] 1.07–1.15), male sex (*P* = 0.003; OR 3.29, 95% CI 1.49–7.40), BMI (*P* = 0.009; OR 1.20, 95% CI 1.05–1.38), hypertension (*P* = 0.021; OR 3.02, 95% CI 1.25–8.46), smoking (*P* = 0.005; OR 4.18, 95% CI 1.65–12.80), and open-type atrophy (*P* < 0.001; OR 2.65, 95% CI 1.86–3.98) as significant risk factors for gastric neoplasms compared to HCs (Table 1). Only advanced age differed significantly between the GA and GC groups (*P* = 0.038), suggesting that clinical variables alone are insufficient to effectively distinguish between these two neoplastic stages.

### Gastric microbiome dysbiosis associated with GC progression

**Fig. 1:**
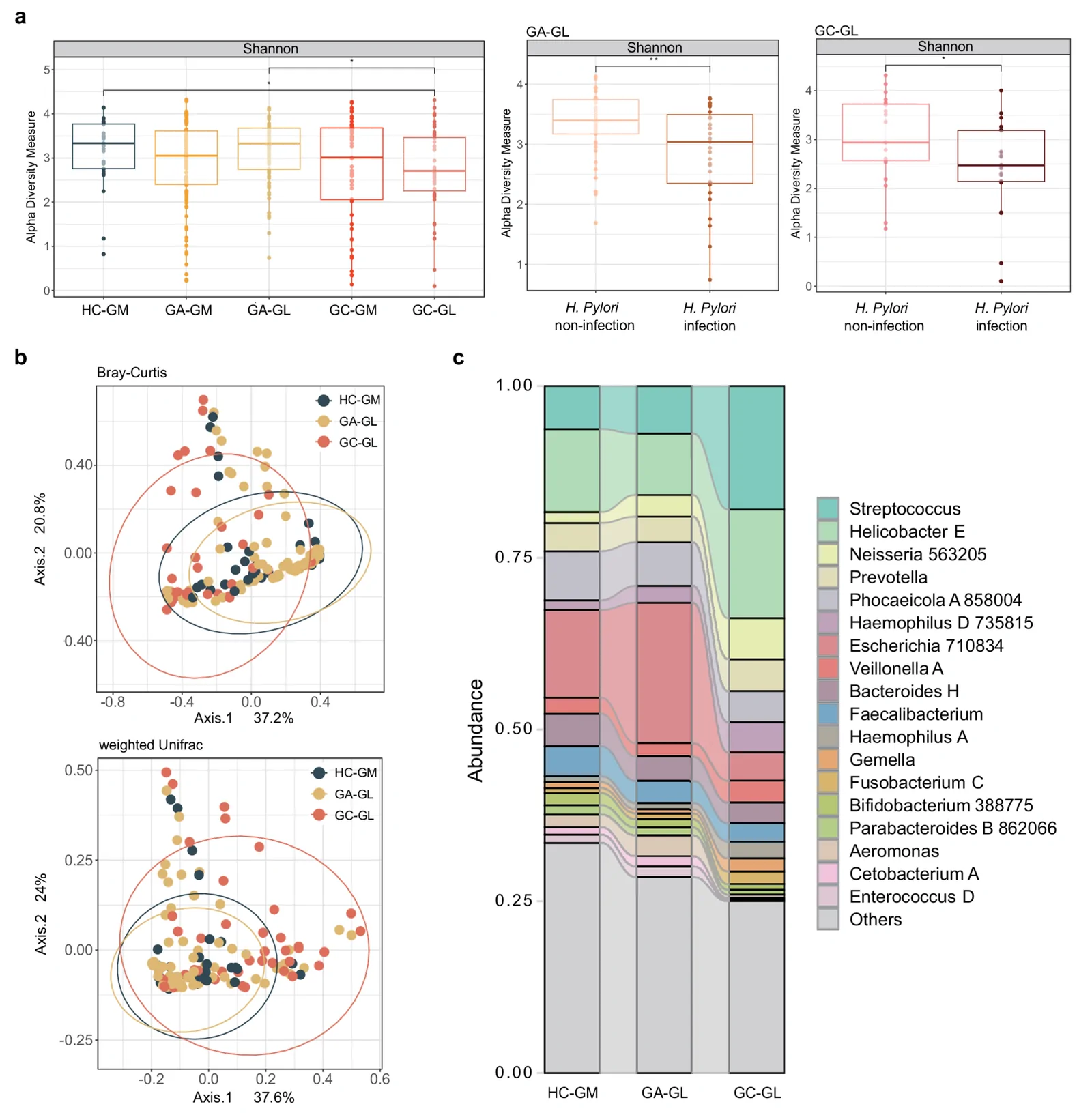
Microbial diversity and taxonomic composition in gastric biopsy samples. (**a**) Comparison of alpha diversity (Shannon index) across sample groups (left panel). The impact of *H. pylori* infection on alpha diversity is shown for GA-GL (middle panel) and GC-GL (right panel). (**b**) Principal Coordinate Analysis (PCoA) plots illustrating beta diversity based on Bray-Curtis dissimilarity and weighted UniFrac distance. Shaded areas represent 95% confidence ellipses for each group based on a t-distribution. (**c**) Relative abundance of bacterial taxa at the genus level in HC-GM, GA-GL, and GC-GL. HC-GM, healthy control gastric mucosa; GA-GM, gastric adenoma adjacent non-lesional mucosa; GA-GL, gastric adenoma lesion; GC-GM, gastric carcinoma adjacent non-lesional mucosa; GC-GL, gastric carcinoma lesion

After denoising and quality control, sequencing yielded 29.1 million reads and 54,456 ASVs from 522 samples. Specifically, we obtained 99 GM, 80 GL, and 126 °C samples from 129 patients with GA; 51 GM, 40 GL, and 68 °C samples from 73 patients with GC; and 29 GM and 29 °C samples from HCs (Supplementary Fig. S1). Alpha diversity remained stable in the oral cavity and non-lesional mucosa across disease stages (Supplementary Fig. S2). However, alpha diversity in lesional mucosa was significantly lower in GC patients (GC-GL) compared to both GA patients (GA-GL; *P* = 0.015) and HCs (HC-GM; *P* = 0.027) (Fig. 1a). *H. pylori* infection was associated with significantly reduced alpha diversity in lesional mucosa samples from both GA (*P* = 0.008) and GC (*P* = 0.049) patients (Fig. 1a), as well as in adjacent non-lesional mucosa (*P* < 0.001; Supplementary Fig. S2c).

Beta diversity analysis demonstrated distinct clustering of GC-GL samples, which were clearly separated from HC-GM and GA-GL samples (Fig. 1b). Taxonomic profiling revealed that *Bacteroides* and *Escherichia* were abundant in HC-GM and GA-GL but depleted in GC-GL (Fig. 1c). Conversely, *Streptococcus*, *Helicobacter*, *Neisseria*, and *Haemophilus* were more abundant in GC-GL. These results indicate that microbial dysbiosis initiates with *H. pylori* infection prior to the adenoma stage and undergoes distinct compositional shifts during the progression from GA to GC.

### Microbial genera associated with GC progression

**Fig. 2:**
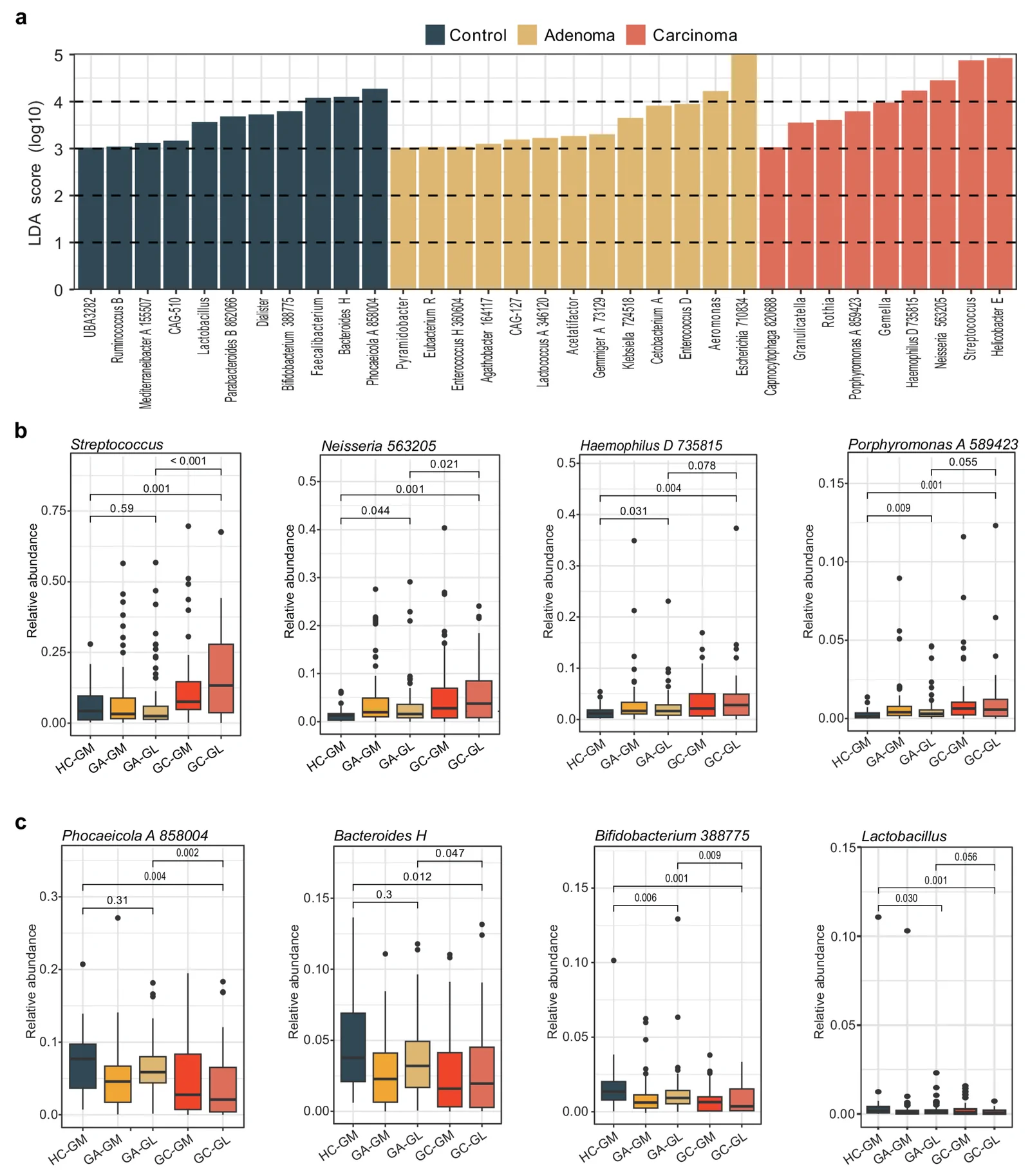
Taxonomic changes in the gastric microbiome during gastric cancer progression. (**a**) LEfSe analysis of gastric microbiota among HC-GM, GA-GL and GC-GL groups. A total of 33 genera showed LDA scores ≥ 3.0. (**b-c**) Boxplots showing the relative abundances of representative genera. *P*-values were calculated using the Wilcoxon rank-sum test

LEfSe analysis identified 33 discriminatory genera with an LDA score ≥ 3 (Fig. 2a). Among these, eight genera, including *Helicobacter*, *Streptococcus*, *Neisseria*, *Haemophilus*, *Gemella*, *Porphyromonas*, *Rothia*, and *Granulicatella*, were enriched in GC-GL (Fig. 2b). Conversely, 21 genera, such as *Phocaeicola*, *Bacteroides*, *Faecalibacterium*, and *Bifidobacterium*, were significantly depleted (Fig. 2c and Supplementary Fig. S3). Notably, the abundance of these genera in GC-GM did not differ significantly from that in GC-GL, suggesting that dysbiosis in GC is not limited to the tumor site. In the GA-GL group, *Escherichia* and *Aeromonas* were specifically enriched (Supplementary Fig. S3). Furthermore, *Neisseria*, *Haemophilus*, and *Porphyromonas* exhibited increased abundance in both GA-GL and GC-GL compared to HC-GM, whereas *Bifidobacterium* and *Lactobacillus* were decreased (Fig. 2b–c). Of note, *Neisseria* abundance showed a gradual increase, while *Bifidobacterium* progressively decreased during the transition from GA to GC. In the multivariate analysis, several key genera retained their significance after adjusting for clinical variables (Supplementary Table S2). Notably, *Streptococcus* and *Neisseria* were independently enriched in GC-GL, whereas taxa like *Phocaeicola*, *Bacteroides*, and *Bifidobacterium* remained significantly depleted, indicating the independent role of the gastric microbiome in cancer progression. Collectively, these data demonstrate distinct taxonomic shifts in the gastric microbiome throughout GC progression.

### Association between microbial genera and clinicopathologic characteristics

**Fig. 3:**
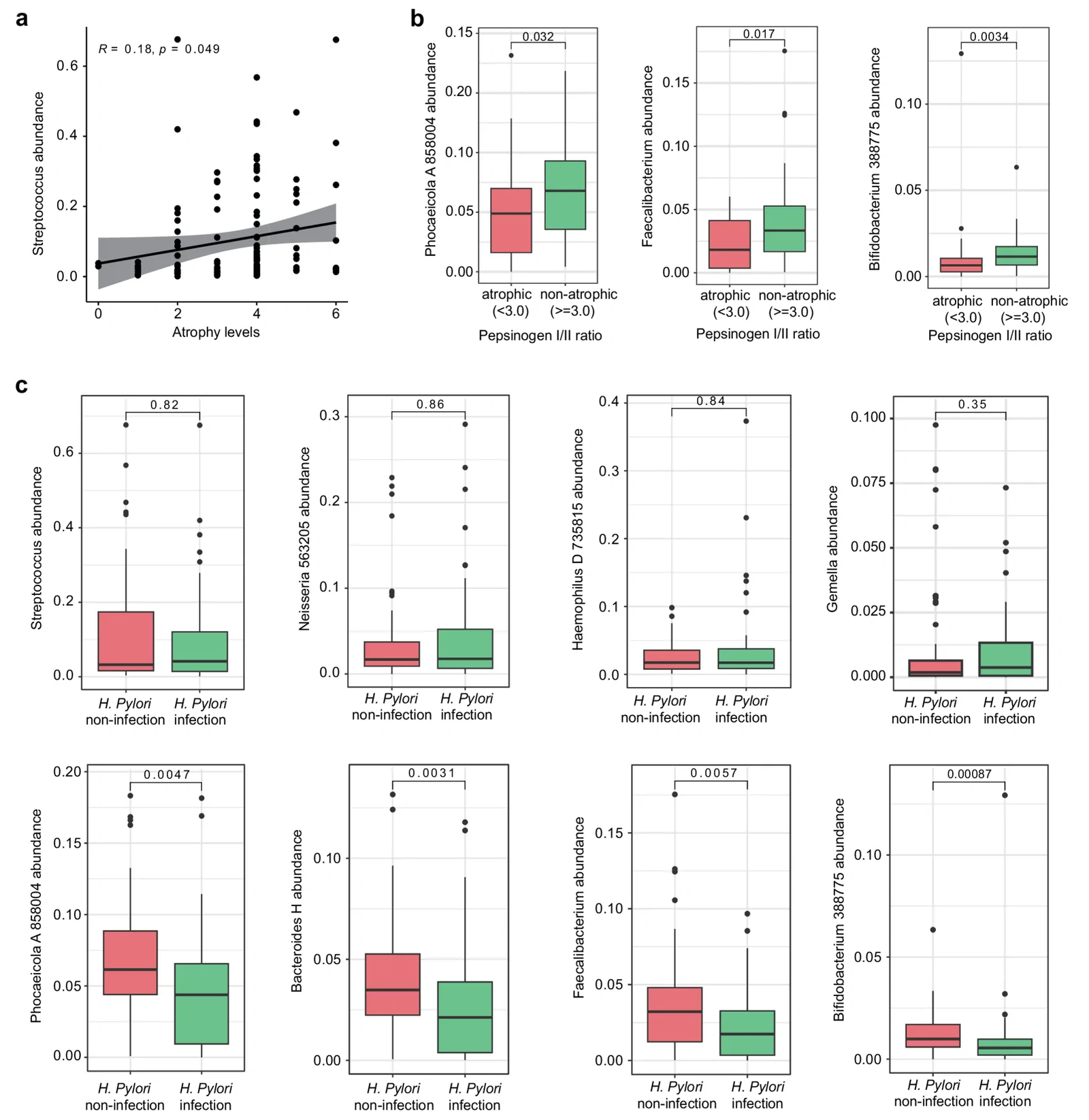
Association between clinical factors and microbial abundance in gastric neoplasms. (**a**) Correlation between *Streptococcus* abundance and mucosal atrophy severity according to the Kimura-Takemoto classification (0–3: No atrophy to Closed type; 4–6: Open type). The correlation was assessed using Spearman’s rank test. (**b**) Differential abundance of genera in gastric neoplasms stratified by serum pepsinogen I/II ratio (< 3 vs. ≥ 3). (**c**) Comparison of key genera abundance based on *H. pylori* infection status. For (**b**) and (**c**), *P*-values were determined using the Wilcoxon rank-sum test

Spearman’s correlation analysis was performed to examine the association between the 33 discriminatory genera identified by LEfSe and clinical GC risk factors. *Streptococcus* abundance in GL samples showed a weak but statistically significant correlation with the endoscopic scoring of mucosal atrophy severity (Kimura–Takemoto classification, rho = 0.18, *P* = 0.049) (Fig. 3a). When stratified by the serum pepsinogen I/II ratio, genera such as *Phocaeicola* (*P* = 0.032), *Faecalibacterium* (*P* = 0.017), and *Bifidobacterium* (*P* = 0.003) were significantly enriched in the non-atrophic group (pepsinogen I/II ratio ≥ 3) compared to the atrophic group (pepsinogen I/II ratio < 3) (Fig. 3b). With regard to *H. pylori* status, GL-enriched genera showed no significant association with infection; however, genera depleted in GL (*Phocaeicola*, *Bacteroides*, *Faecalibacterium*, and *Bifidobacterium*) were significantly less abundant in *H. pylori*-infected patients compared to uninfected patients (Fig. 3c).

### Predictive modeling of gastric neoplasms

**Fig. 4:**
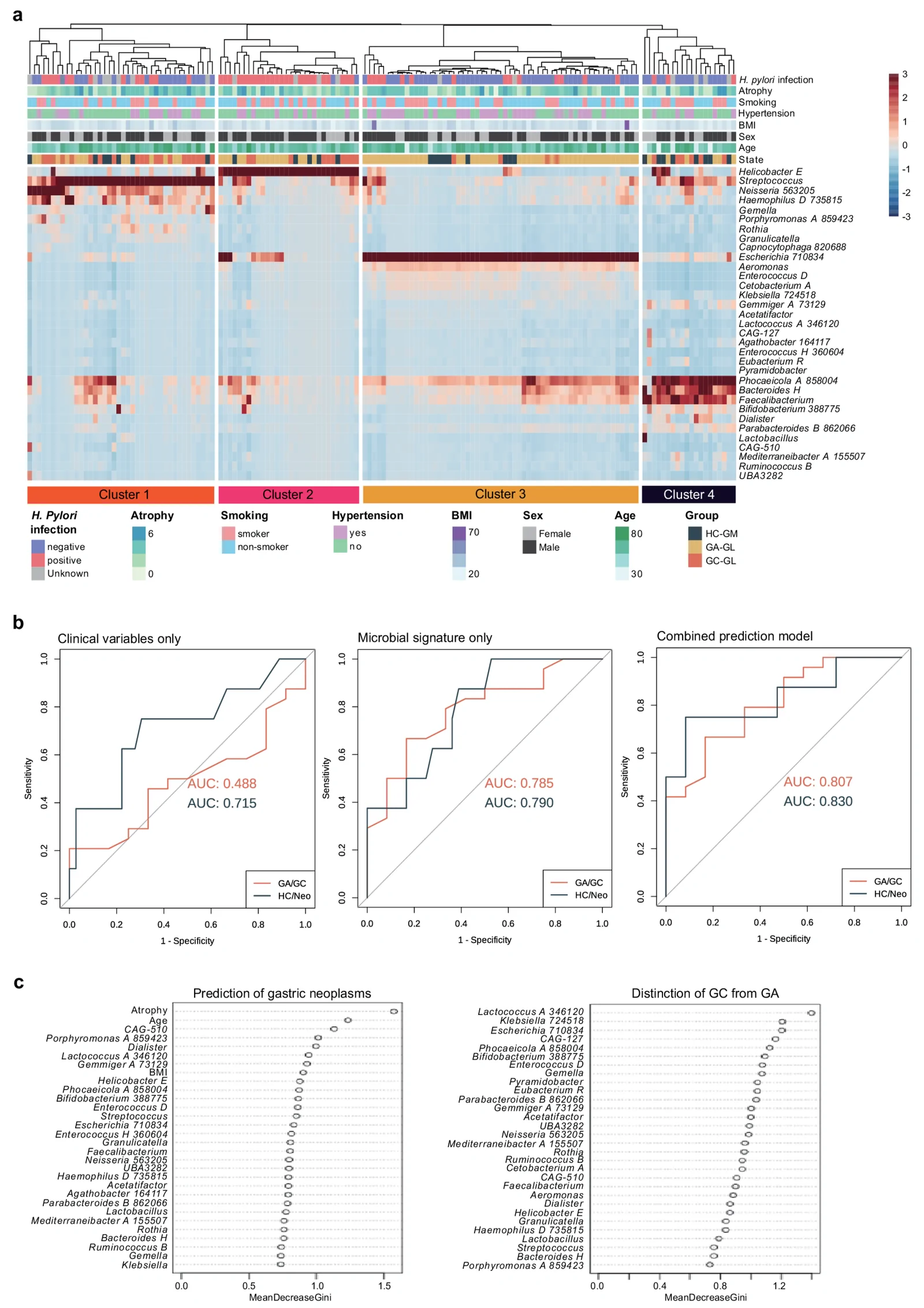
Random Forest-based discrimination of gastric neoplasms using microbial signatures and clinical variables. (**a**) Heatmap showing hierarchical clustering of gastric samples based on the 33 discriminatory genera (LDA score > 3.0). Samples are annotated with clinical factors, including *H. pylori* infection status, mucosal atrophy grade, smoking status, BMI, sex, age, and lesion type (HC-GM, GA-GL, and GC-GL). Four distinct clusters were identified: a GC-dominant cluster, an *H. pylori*-dominant cluster, a GA-dominant cluster, and a mixed cluster. (**b**) Performance evaluation of the Random Forest models for discriminating gastric lesions. ROC curves compare three models based on: (1) clinical variables alone, (2) microbial signature alone, and (3) a combination of both. The blue line represents the model distinguishing gastric neoplasms (GA and GC) from healthy controls, while the red line represents the model distinguishing GC from GA. (**c**) Variable importance plots based on the Mean Decrease Gini index. The left panel displays the top 30 features contributing to the prediction of gastric neoplasms, while the right panel identifies the top 30 features distinguishing GC from GA

Unsupervised hierarchical clustering of the 33 genera revealed four distinct clusters: Cluster 1 (GC-dominant; 50.0% [20/40] of GC-GL samples), Cluster 2 (*Helicobacter*-dominant), Cluster 3 (GA-dominant; 57.5% [46/80] of GA-GL samples), and Cluster 4 (mixed) (Fig. 4a). The genera *Streptococcus*,* Neisseria*, and *Haemophilus* were enriched in Cluster 1, whereas *Aeromonas* and *Escherichia* were enriched in Cluster 3, consistent with our LEfSe analysis. Cluster 4 was characterized by the genera *Phocaeicola*, *Bacteroides*, *Faecalibacterium*, and *Bifidobacterium*, which were identified as abundant in HC-GM samples based on LEfSe analysis. Of note, Cluster 4 accounted for 27.6% of the HC-GM samples (8/29), which was higher than that of GA-GL (7.5%; 6/80) and GC-GL (15.0%; 6/40).

To evaluate diagnostic utility, random forest models were constructed using LOOCV to integrate microbial and clinical data. The combined prediction model incorporating 33 microbial genera identified by LEfSe and six clinical variables (age, sex, BMI, hypertension, smoking, and atrophy) achieved a superior AUC of 0.830 in distinguishing gastric neoplasms from HC, outperforming models based solely on microbial signatures (AUC = 0.790) or clinical variables (AUC = 0.715) (Fig. 4b). At a threshold of 0.743, the model yielded a sensitivity of 91.7%, specificity of 75.0%, and accuracy of 88.6%. Furthermore, in differentiating GC from GA, the combined model showed higher discriminatory power (AUC = 0.807) than the model using clinical variables alone (AUC = 0.488) (Fig. 4b). The sensitivity, specificity, and accuracy of the combined prediction model for distinguishing GA and GC were 83.3%, 66.7%, and 72.2%, respectively, based on a discriminant score threshold of 0.308. Feature importance analysis identified mucosal atrophy, age, and the genera *CAG-510*, *Porphyromonas*, and *Dialister* as the primary contributors to the neoplasm prediction model, whereas *Lactococcus*, *Klebsiella*, and *Escherichia* were key features for distinguishing GC from GA (Fig. 4c).

### Predicted functional alterations of the gastric microbiome

**Fig. 5:**
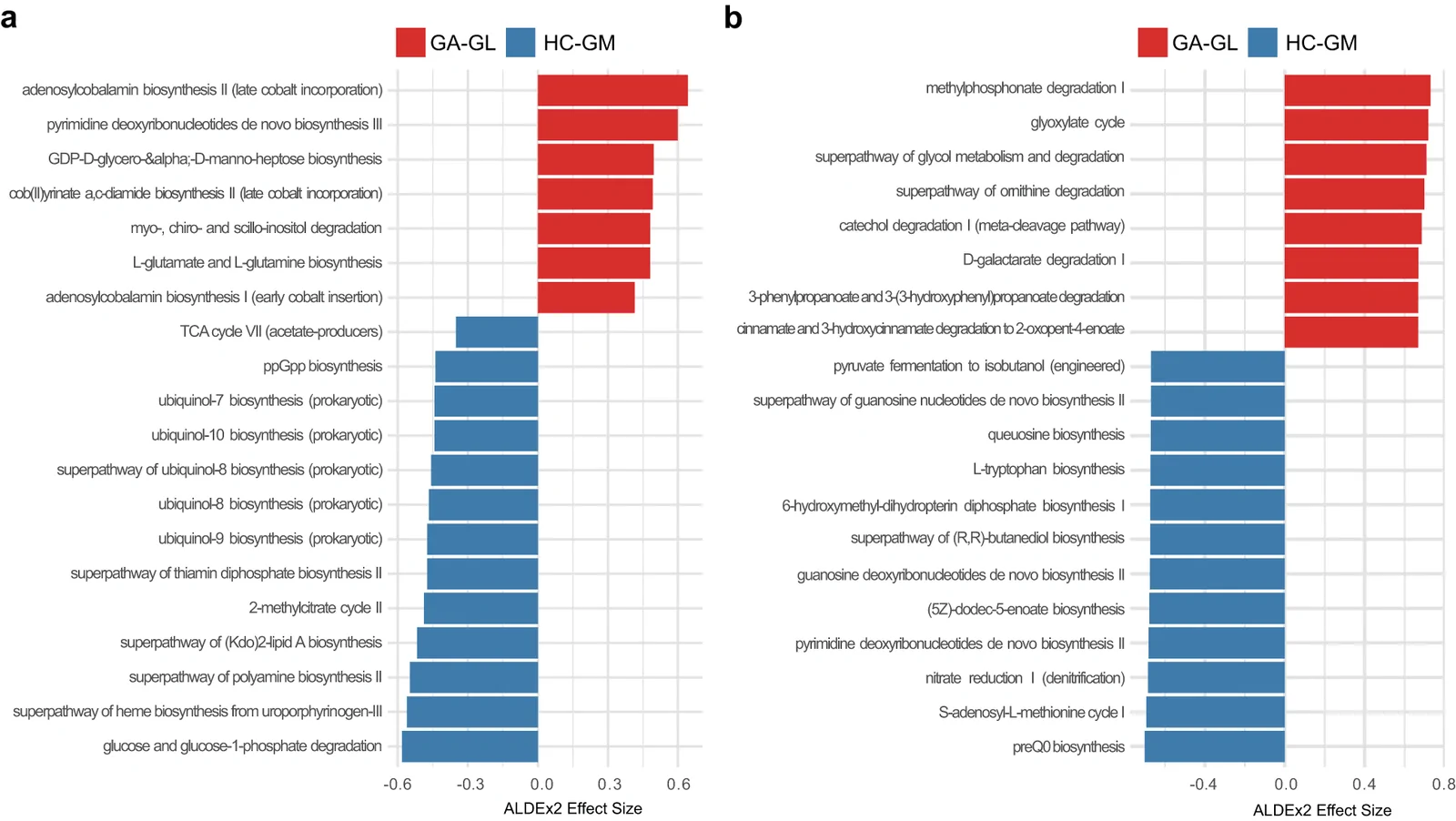
Predicted functional metabolic profiles of the gastric microbiome in GC and GA lesions compared to HC mucosa. Functional prediction was performed using PICRUSt2 based on ASVs corresponding to the 33 discriminatory genera identified by LEfSe (LDA score > 3.0). The figure displays the top 30 differentially abundant MetaCyc pathways between lesion tissues and healthy control mucosa. (**a**) Comparison between GA-GL and HC-GM, showing pathways with an absolute effect size ≥ 0.3. (**b**) Comparison between GC-GL and HC-GM, showing pathways with an absolute effect size ≥ 0.3

Functional prediction using PICRUSt2 suggested potential metabolic shifts. Compared to HC-GM, 20 MetaCyc pathways were differentially abundant in each of the GA-GL and GC-GL groups. In GA-GL, seven pathways, such as pyrimidine deoxyribonucleotides *de novo* biosynthesis III, were enriched (effect size > 0.3), whereas 13 pathways, such as glucose and glucose-1-phosphate degradation, were depleted (Fig. 5a). In GC-GL, eight pathways, including the superpathway of glycol metabolism and degradation, were increased (Fig. 5b). Conversely, 12 pathways, including pyrimidine deoxyribonucleotides *de novo* biosynthesis II, were decreased.

## Discussion

This study provides a comprehensive analysis of the multifocal gastric microbiome in patients with GA and GC, incorporating comparisons with healthy individuals. Using 16 S rDNA sequencing of paired OC, GM, and GL samples, we identified progressive and distinct alterations in microbial diversity, taxonomic composition, and functional pathways associated with GC. *H. pylori* infection was associated with reduced alpha diversity across all disease groups, implicating it as the initial driver of dysbiosis. Moreover, taxonomic shifts were observed not only in neoplastic lesions but also in the adjacent normal mucosa, suggesting a broader field effect of microbial dysbiosis. Importantly, our machine learning-based prediction models emphasize the diagnostic potential of integrating microbiome data with clinical variables.

Our findings revealed a significant decrease in alpha diversity in GC-GL samples compared to GA-GL and HC-GM samples, particularly in *H. pylori*-infected individuals. According to Correa’s cascade, *H. pylori* initiates gastric carcinogenesis through chronic inflammation, parietal cell loss, mucosal atrophy, and intestinal metaplasia [20]. However, accumulating evidence indicates that *H. pylori* infection alone is insufficient to drive GC development, and its relative abundance tends to decline as metaplasia progresses [7]. *H. pylori*-induced inflammation leads to the depletion of acid-secreting parietal cells and a subsequent reduction in intragastric acidity [21], which in turn facilitates the colonization of non-*H. pylori* bacteria. These secondary microbial shifts contribute to dysbiosis and are hypothesized to promote tumorigenesis through mechanisms such as the production of carcinogenic metabolites (e.g., *N*-nitroso compounds and lactate), immune modulation, oxidative stress, DNA damage, and epithelial–mesenchymal transition [22]. These findings emphasize the necessity of characterizing the non-*H. pylori* community to fully understand the microbial mechanisms involved in the later stages of GC development.

LEfSe analysis revealed distinct microbial signatures associated with different stages of gastric neoplasms. Notably, the genera *Streptococcus*, *Neisseria*, and *Haemophilus* were significantly enriched in GC-GL, whereas genera such as *Bifidobacterium*, *Phocaeicola*, and *Faecalibacterium* were markedly depleted. These findings align with previous reports that pro-inflammatory or opportunistic genera increase during tumorigenesis, while anti-inflammatory genera decline [14, 23]. Specifically, *Streptococcus anginosus* has been shown to promote inflammation and epithelial damage in gastric tissues, facilitating neoplastic progression [12]. Similarly, *Neisseria* may contribute to gastric cancer development through genotoxin production [24] and nitrosamine formation [25, 26]. *Haemophilus*, which has been associated with inflammation and metaplasia [27, 28], has also been identified as a central hub in gastric cancer networks [29]. Additionally, *Porphyromonas* may promote carcinogenesis by inhibiting epithelial cell apoptosis [30]. Conversely, the depletion of *Bifidobacterium* suggests a loss of its protective roles, particularly in immune regulation and the maintenance of epithelial integrity [31].

Notably, our multivariate analysis confirmed that key genera retain independent associations with gastric neoplasms after adjusting for clinical variables. In contrast, several oral-associated taxa (e.g. *Haemophilus* and *Rothia*) and *Helicobacter* lost statistical significance. This attenuation suggests that their enrichment may be secondary to clinical confounders, such as aging and smoking, which facilitate the passive translocation of oral commensals to the stomach. Furthermore, the loss of significance for *Helicobacter* likely reflects its strong collinearity with mucosal atrophy. These findings emphasize that while univariate shifts reflect a dysbiotic environment, integrating clinical variables is essential to filter out passenger microbes and identify true driver signatures. This rationale aligns with the superior performance of our integrated random forest model. By combining 33 discriminatory genera with six clinical variables, the model substantially exceeded the performance of models using microbiome or clinical data alone [32]. This synergy demonstrates that microbiome signatures continue to evolve during the premalignant-to-malignant transition and can provide incremental diagnostic value. Collectively, these findings highlight the potential of microbiome-informed machine learning as valuable adjunctive tools for early detection and risk stratification in clinical practice.

While the oral microbiota has been implicated in GC carcinogenesis [11, 12, 33, 34], we observed no significant compositional changes in the oral cavity across disease stages. However, we found elevated *Streptococcus* abundance in GL from both GA and GC patients compared to HC-GM, which positively correlated with mucosal atrophy severity and increased with atrophy progression. This is consistent with the proposed links between *Streptococcus* and inflammatory carcinogenic mechanisms [35–37]. This finding suggests a potential translocation of oral commensals to the gastric niche, favored by the changing microenvironment (e.g., hypochlorhydria), rather than a distinct compositional shift within the oral cavity itself.

Predicted pathway enrichment analysis suggests potential progressive metabolic reprogramming during gastric carcinogenesis, characterized by distinct functional categories. The transition from gastric adenoma to carcinoma might involve a continuous metabolic and microenvironmental shift. In the GA stage, we observed a decrease in predicted glucose degradation pathways compared to healthy individuals, suggesting a unique metabolic adaptation that precedes the classic Warburg effect [38]. In contrast, the progression to carcinoma is marked by a significant increase in the superpathway of glycol metabolism and degradation, a hallmark of cancer that reflects high energy and biomass demands [39]. Furthermore, while adenoma exhibits an enrichment in pathways for the *de novo* biosynthesis of DNA components, the carcinoma stage shows a decrease in this pathway. This observation, coupled with the sustained demand for building blocks like L-glutamate and L-glutamine, suggests a shift towards more energy-efficient nucleotide salvage pathways in advanced cancer [40]. Finally, our data are compatible with the increasingly recognized role of the microbiome in this progression. The early increase in GDP-D-glycero-α-D-manno-heptose biosynthesis in GA is consistent with the involvement of Gram-negative bacteria. As the disease advances, a more diverse range of microbial-related degradation pathways becomes prominent, suggesting an active microbial ecosystem that likely contributes to malignancy [9, 41].

In interpreting the GC-enriched genera identified in our cohort, it is important to distinguish whether specific genera act as drivers of carcinogenesis or represent disease-associated markers resulting from ecological adaptation to the altered gastric environment. Among the genera we identified, Streptococcus (particularly S. anginosus) has the strongest mechanistic support, as recent studies in murine models have shown it can directly induce gastric inflammation, atrophy, and tumorigenesis [12]. Neisseria and Porphyromonas, although biologically plausible drivers via genotoxin production and apoptosis inhibition, have primarily been characterized in oral or colorectal contexts, and their causal role in the stomach remains to be fully elucidated. In contrast, depleted genera such as Bifidobacterium, Faecalibacterium, and Phocaeicola likely represent a collateral loss of commensal taxa under hypochlorhydric conditions, although their depletion may secondarily contribute to a permissive pro-inflammatory microenvironment. Therefore, while several of our findings are consistent with a driver role for selected oral-origin pathobionts, we conservatively interpret the majority of the dysbiotic signature as disease-associated rather than causal until further functional validation is performed.

This study has several strengths. It represents the largest cohort to date analyzing the gastric microbiome across multiple stages of GC progression. Using matched samples from multiple gastric sites and the oral cavity allowed us to capture spatial microbial dynamics. Our rigorous sampling protocols minimized the risk of contamination. However, our study also has some limitations. First, as the study focused on early-stage GC, generalizability to advanced cases may be limited. Second, all participants were Korean, which may restrict extrapolation to other populations. Incomplete clinical data on *H. pylori* status in some healthy participants may have impacted the predictive models’ performance. Third, since feature selection was conducted using the full cohort prior to the data split, the reported AUC should be interpreted as an optimistic estimation of diagnostic performance. Furthermore, our functional analysis was based on PICRUSt2, which provides inferred rather than directly measured functional profiles. Finally, although we identified GC-specific microbial genera and integrated them into a predictive model, functional validation of these genera was not performed. Therefore, the biological relevance of these microbes in gastric carcinogenesis remains hypothesis-generating and speculative.

In conclusion, our findings highlight stage-specific changes in the gastric microbiome during GC development. These alterations include both compositional and functional changes that extend beyond *H. pylori* infection. GC-specific microbial genera and metabolic pathways suggest new directions for researching microbial contributions to tumor metabolism and host-microbe interactions, which could be leveraged for therapeutic or preventive strategies against GC. In practice, integrating microbial signatures and clinical features could complement current diagnostic approaches, such as endoscopy and histology.

## Supplementary Information

Supplementary Material 1.

## Data Availability

Raw sequencing reads have been deposited in the Sequence Read Archive under the accession number PRJNA1252285.
