# Supplementary material for: Integrated analysis of gastric microbiome and clinical features for the diagnosis of gastric neoplasms
Source: Gut Pathog. 2026 Jun 12;18:68. doi: 10.1186/s13099-026-00843-3 (PMC13520268; doi:10.1186/s13099-026-00843-3)
Supplement: Supplementary file 1 [file 13099_2026_843_MOESM1_ESM.pdf]

Supplementary Materials

Table of Contents

Supplementary Methods…………………………………………………………....page 1

Supplementary Figure 1……………………………………………………………page 5

Supplementary Figure 2……………………………………………………………page 6

Supplementary Figure 3……………………………………………………………page 7

Supplementary Table 1…………………………………………………..…………page 9

Supplementary Table 2…………………………………………………..…………page 10

Supplementary References……………………………………………………...page 12

Supplementary Methods

Bacterial 16S ribosomal DNA sequencing

The samples were stored at −80°C until DNA extraction. Genomic DNA was extracted from buccal swabs and tissue samples using the Maxwell RSC PureFood GMO and Authentication Kit (Promega, Madison, WI, USA). DNA concentrations were measured using a NanoDrop spectrophotometer (Thermo Fisher Scientific, Waltham, MA, USA) and quantified using the QuantiFluor ONE dsDNA System (Promega). DNA samples were stored at -20°C until library preparation.

The hypervariable V3–V4 region of the bacterial 16S rRNA gene was amplified by polymerase chain reaction (PCR) using the 16S metagenomics primer set (Forward: 5′-TCGTCGGCAGCGTCAGATGTGTATAAGAGACAGCCTACGGGNGGCWGCAG-3’, Reverse: 5′-GTCTCGTGGGCTCGGAGATGTGTATAAGAGACAGGACTACHVGGGTAT-CTAATCC-3′). PCR products were purified using Agencourt AMPure XP magnetic beads (Beckman Coulter, Brea, CA, USA). Following purification, index PCR was performed to attach Illumina Nextera barcodes (Illumina, San Diego, CA, USA) using i5 forward and i7 reverse primers. The size and quality of the purified amplicons were determined using an Agilent 2100 Bioanalyzer (Agilent Technologies, Santa Clara, CA, USA), and the final products were quantified using a Quantus fluorometer (Promega). The pooled libraries were sequenced on the Illumina MiSeq platform using a MiSeq Reagents Kit v3 (Illumina). Raw sequencing reads have been deposited in the NCBI Sequence Read Archive (SRA) under accession number PRJNA1065147.

Analysis of bacterial 16S ribosomal DNA sequencing data

All sequencing data were processed using QIIME 2 (version 2023.7.0) [1]. Raw sequences were demultiplexed, quality filtered, and denoised using the DADA2 plugin [2]. For phylogeny

analysis, a phylogenetic tree was constructed using the phylogeny plugin with MAFFT alignment and FastTree. Taxonomy was assigned to amplicon sequence variants (ASVs) using the feature-classifier plugin trained on a Greengenes2 V3–V4 custom classifier. This classifier was generated using the extract-reads and fit-classifier-naive-bayes functions based on the Greengenes2 database (2022.10 release), which unifies genomic and 16S rRNA databases into a consistent phylogenetic framework [3].

A phyloseq object was generated from QIIME 2 artifacts using the qiime2R package (v0.99.6). Non-informative ASVs, such as those unassigned at the genus level, were filtered out using the phyloseq package [4]. Alpha diversity (Shannon index) was calculated based on the genus-level abundance table for each disease group. Beta diversity was assessed using Bray-Curtis dissimilarity and weighted UniFrac distance matrices, visualized via Principal Coordinate Analysis (PCoA) plots. For visualization of group clusters, 95% confidence ellipses were calculated and plotted assuming a t-distribution using the "stat_ellipse" function in R.

Differential abundance analysis was conducted using the Linear Discriminant Analysis Effect Size (LEfSe) algorithm [5]. Taxa with a Linear Discriminant Analysis (LDA) score > 3.0 and a Kruskal-Wallis test P-value < 0.05 were considered statistically significant. Correlation analyses were performed and visualized using the corrplot package (https://github.com/taiyun/corrplot) and the ggscatter function from the ggpubr package (https://rpkgs.datanovia.com/ggpubr/). A heatmap visualizing the relative abundance of genera with an LDA score > 3.0 was generated using the pheatmap package (https://github.com/raivokolde/pheatmap/) based on Euclidean distance clustering. For the genus *Helicobacter*, clinical *H. pylori* infection status was used as a substitute in the heatmap visualization.

Functional profiles of the identified sequences were predicted using PICRUSt2 based on MetaCyc pathways [6]. This analysis compared GC lesions with HC mucosa and GA lesions

with HC mucosa, focusing on sequences associated with genera exhibiting an LDA score ≥ 3.0. The predicted functional profiles were visualized using the ggpicrust2 package (v1.7.4) [7].

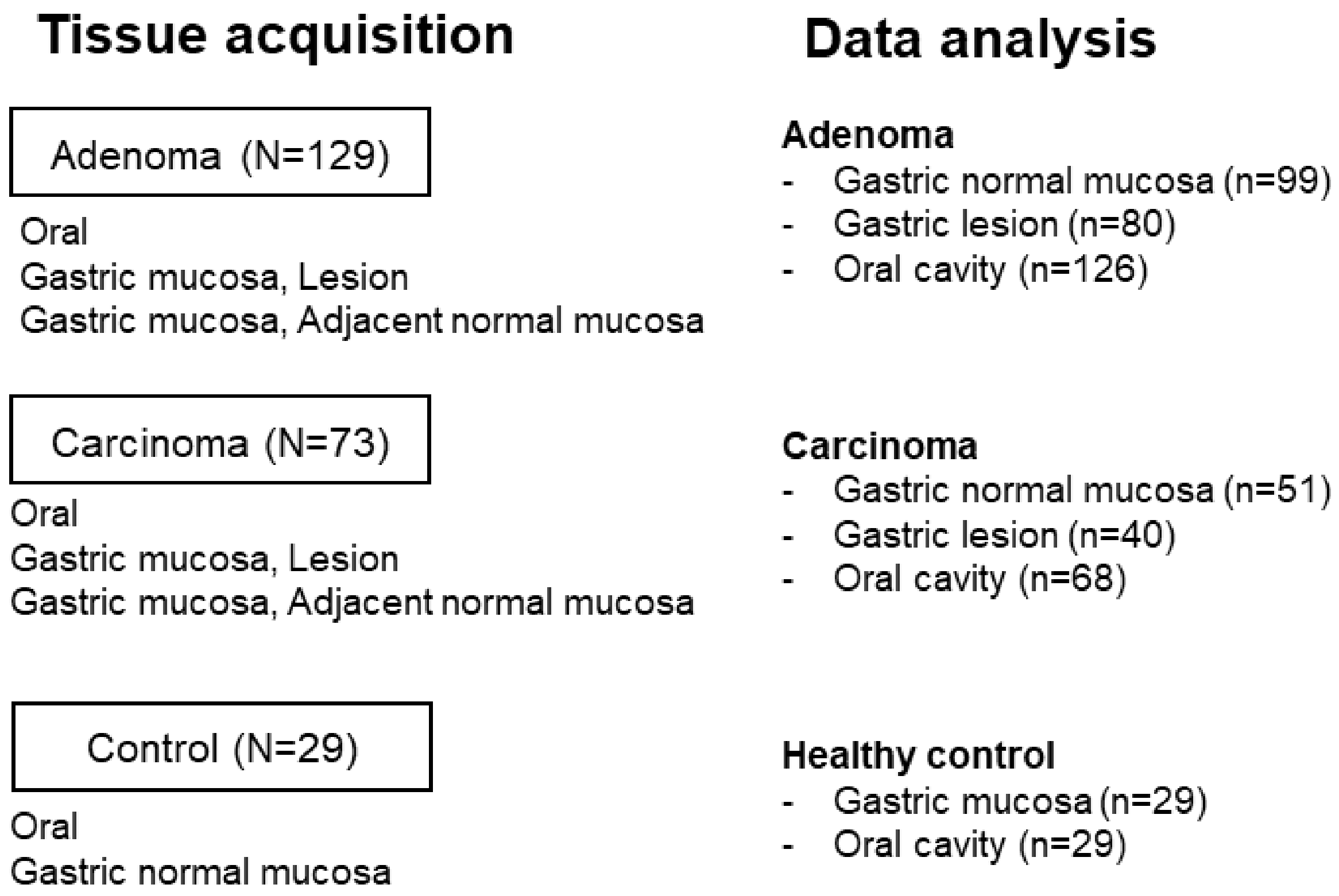

Supplementary Fig. 1. Overview of tissue acquisition and data analysis strategy. This figure illustrates the workflow for tissue sampling and subsequent analysis. The "Tissue acquisition" panel displays the sample sources for the adenoma, carcinoma, and healthy control groups. Samples collected included gastric lesions and adjacent normal mucosa for the patient groups, and normal gastric mucosa for the healthy controls; oral cavity samples were obtained from all groups. The "Data analysis" panel specifies the tissue subsets analyzed for each condition: gastric lesions, adjacent normal mucosa, and oral cavity samples for the adenoma and carcinoma groups, and normal gastric mucosa and oral cavity samples for the healthy controls.

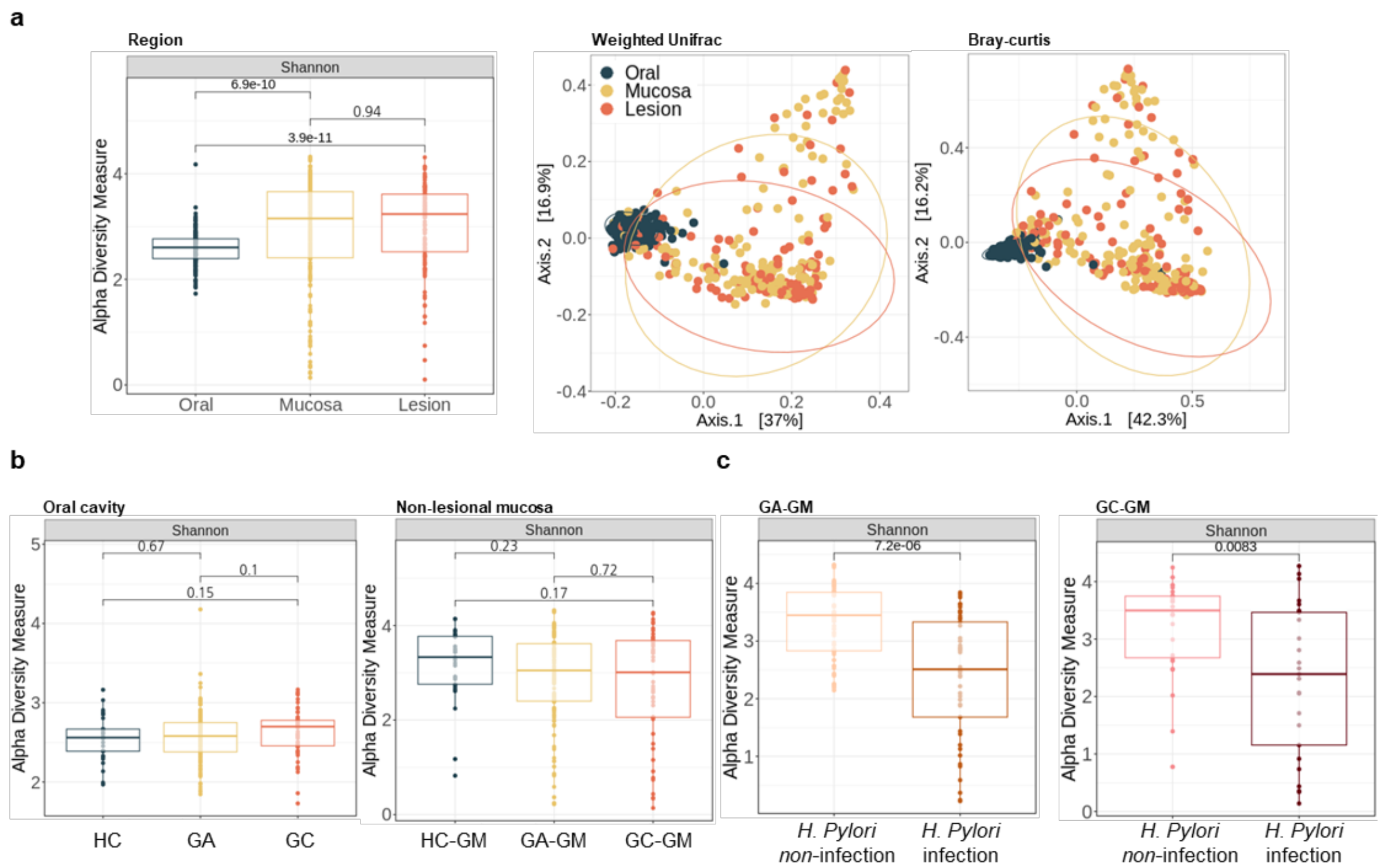

Supplementary Fig. 2. Alpha and beta diversity of microbial communities across different sampling sites in GA, GC, and HC groups. (a) Comparison of alpha diversity among the oral cavity, adjacent non-lesional gastric mucosa, and gastric lesions. Beta diversity was analyzed based on weighted UniFrac distances and Bray-Curtis dissimilarity at the genus level. (b) Comparison of alpha diversity among HC, GA, and GC groups in both the oral cavity and adjacent non-lesional mucosa. (c) The impact of *H. pylori* infection on alpha diversity within the adjacent non-lesional mucosa of gastric adenoma and carcinoma groups.

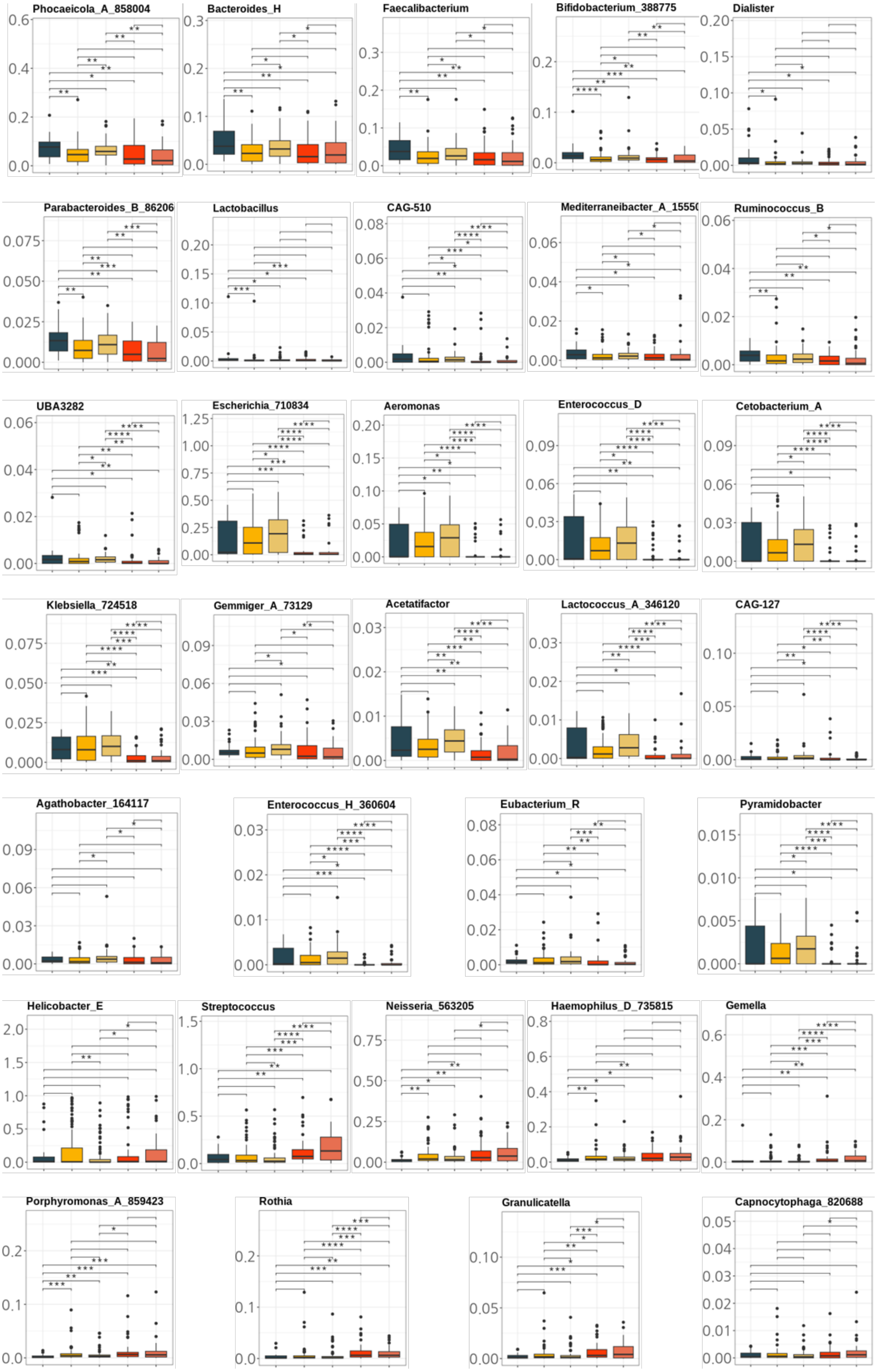
Phocaeicola_A_858004
Bacteroides_H
Faecalibacterium
Bifidobacterium_388775
Dialister
Parabacteroides_B_86206
Lactobacillus
CAG-510
Mediterraneibacter_A_1555
Ruminococcus_B
UBA3282
Escherichia_710834
Aeromonas
Enterococcus_D
Cetobacterium_A
Klebsiella_724518
Gemmiger_A_73129
Acetatifactor
Lactococcus_A_346120
CAG-127
Agathobacter_164117
Enterococcus_H_360604
Eubacterium_R
Pyramidobacter
Helicobacter_E
Streptococcus
Neisseria_563205
Haemophilus_D_735815
Gemella
Porphyromonas_A_859423
Rothia
Granulicatella
Capnocytophaga_820688

Supplementary Fig. 3. Boxplots of relative abundances of genera with an LDA score greater than 3. P-values were calculated using the Wilcoxon rank-sum test. Significance is indicated as follows: **** ($P \leq 0.0001$), *** ($P \leq 0.001$), ** ($P \leq 0.01$), * ($P \leq 0.05$). Non-significant comparisons (ns) are not displayed.

Supplementary Table 1. Baseline clinicopathologic characteristics of the study population

	Adenoma (N=129)	Carcinoma (N=73)	Healthy Control (N=29)
**Age (mean ± SD)**	65.0 ± 10.1	68.0 ± 9.3	51.8 ± 14.8
**Sex, male (n, %)**	94 (72.9)	53 (72.6)	13 (44.8)
**BMI (kg/m^2, mean ± SD)**	25.4 ± 6.4	24.2 ± 2.6	22.9 ± 2.7
**Hypertension, yes (n, %)**	61 (47.7)	27 (37.0)	6 (20.7)
**Diabetes, yes (n, %)**	25 (19.4)	12 (16.4)	1 (3.4)
**Dyslipidemia, yes (n, %)**	47 (36.4)	23 (31.5)	8 (27.6)
**Smoking (n, %)**			
**Current smoker (n, %)**	9 (7.0)	7 (9.6)	1 (3.4)
**Ex-smoker (n, %)**	45 (34.9)	33 (45.2)	4 (13.8)
**Never smoker (n, %)**	75 (58.1)	33 (45.2)	24 (82.8)
**Mucosal atrophy (n, %)**			
**No atrophy**	2 (1.7)	1 (1.4)	0 (0.0)
**Closed-I**	9 (7.0)	2 (2.7)	17 (58.6)
**Closed-II**	28 (21.7)	16 (21.9)	4 (13.8)
**Closed-III**	14 (10.9)	14 (19.2)	5 (17.2)
**Open-I**	56 (43.4)	30 (41.1)	2 (6.9)
**Open-II**	14 (10.9)	6 (8.2)	1 (3.4)
**Open-III**	6 (4.7)	4 (5.5)	0 (0.0)
**Pepsinogen I/II ratio (n, %)**			
**Not checked**	39 (30.2)	26 (35.6)	29 (100)
**Decreased (<3.0)**	41 (31.8)	23 (31.5)	-
**Normal (>=3.0)**	49 (38.0)	24 (32.9)	-
***H. pylori* status (n, %)**			
**Unknown**			17 (58.6)
***H. pylori* positive**	53 (41.1)	36 (49.3)	3 (10.3)
***H. pylori* negative**	76 (58.9)	37 (50.7)	9 (31.0)
**Location of the lesion (n, %)**			
**Lower third**	64 (49.6)	51 (69.9)	
**Mid third**	54 (41.9)	15 (20.5)	
**Upper third**	11 (8.5)	7 (9.6)	

Supplementary Table 2. Multivariable linear regression analysis of group-associated genus-level abundance differences

Genus	Comparison (vs. HC-GM)	Estimate (log10 scale)	Std.Error	Statistic	*P*-value
Helicobacter_E	GA-GL	-0.74365	0.421163	-1.7657	0.07963
	GC-GL	0.192708	0.475117	0.405601	0.68566
Streptococcus	GA-GL	-0.14427	0.146509	-0.98473	0.32645
	GC-GL	0.384865	0.165278	2.328594	0.02131
Neisseria_563205	GA-GL	0.248912	0.170248	1.462062	0.14597
	GC-GL	0.562469	0.192057	2.928648	0.00398
Haemophilus_D_735815	GA-GL	0.093166	0.148633	0.626815	0.53180
	GC-GL	0.311766	0.167674	1.859353	0.06508
Gemella	GA-GL	-0.43764	0.31087	-1.4078	0.16141
	GC-GL	0.440919	0.350694	1.257274	0.21075
Porphyromonas_A_859423	GA-GL	0.394499	0.250932	1.572136	0.11818
	GC-GL	0.537849	0.283078	1.900004	0.05949
Rothia	GA-GL	-0.13086	0.269902	-0.48485	0.62854
	GC-GL	0.325026	0.304478	1.067487	0.28759
Granulicatella	GA-GL	-0.13676	0.325894	-0.41966	0.67538
	GC-GL	0.260896	0.367644	0.709643	0.47911
Capnocytophaga_820688	GA-GL	-0.46411	0.346867	-1.338	0.18307
	GC-GL	0.154901	0.391303	0.395861	0.69281
Escherichia_710834	GA-GL	0.220674	0.238091	0.926848	0.35560
	GC-GL	-1.17511	0.268592	-4.37505	0.00002
Aeromonas	GA-GL	1.237902	0.503011	2.460982	0.01507
	GC-GL	-1.25648	0.567451	-2.21426	0.02843
Enterococcus_D	GA-GL	0.802735	0.483146	1.661476	0.09886
	GC-GL	-1.28227	0.54504	-2.35261	0.02003
Cetobacterium_A	GA-GL	1.353739	0.484158	2.796066	0.00590
	GC-GL	-0.93565	0.546182	-1.71307	0.08891
Klebsiella_724518	GA-GL	-0.20622	0.309174	-0.66699	0.50588
	GC-GL	-1.23981	0.348781	-3.55471	0.00052
Gemmiger_A_73129	GA-GL	-0.18126	0.261419	-0.69335	0.48924
	GC-GL	-0.64722	0.294909	-2.19465	0.02984
Acetatifactor	GA-GL	0.070503	0.307984	0.228916	0.81927
	GC-GL	-1.4255	0.347439	-4.10288	0.00007
Lactococcus_A_346120	GA-GL	0.587882	0.365194	1.609781	0.10970
	GC-GL	-0.97611	0.411978	-2.36932	0.01919
CAG-127	GA-GL	0.443018	0.371082	1.193853	0.23455
	GC-GL	-1.2364	0.418621	-2.95352	0.00369
Agathobacter_164117	GA-GL	0.005623	0.341682	0.016458	0.98689
	GC-GL	-0.81977	0.385454	-2.12676	0.03519

Enterococcus_H_360604	GA-GL	0.351256	0.378897	0.927049	0.35550
	GC-GL	-1.05194	0.427437	-2.46105	0.01507
Eubacterium_R	GA-GL	0.271483	0.347445	0.781368	0.43591
	GC-GL	-0.68393	0.391956	-1.74491	0.08320
Pyramidobacter	GA-GL	0.91916	0.396899	2.315854	0.02202
	GC-GL	-0.70523	0.447745	-1.57508	0.11749
Phocaeicola_A_858004	GA-GL	-0.18353	0.164122	-1.11826	0.26537
	GC-GL	-0.78234	0.185147	-4.2255	0.00004
Bacteroides_H	GA-GL	-0.17472	0.150147	-1.16364	0.24655
	GC-GL	-0.56747	0.169382	-3.35024	0.00104
Faecalibacterium	GA-GL	-0.19871	0.207616	-0.95712	0.34016
	GC-GL	-0.83208	0.234213	-3.55265	0.00052
Bifidobacterium_388775	GA-GL	-0.38761	0.206461	-1.87741	0.06254
	GC-GL	-1.12066	0.232911	-4.81157	0.00000
Dialister	GA-GL	-0.26771	0.236533	-1.13181	0.25965
	GC-GL	-0.68606	0.266834	-2.57111	0.01118
Parabacteroides_B_862066	GA-GL	-0.14234	0.184957	-0.76959	0.44284
	GC-GL	-0.79621	0.208651	-3.81599	0.00020
Lactobacillus	GA-GL	-0.6482	0.313357	-2.06855	0.04043
	GC-GL	-1.2258	0.3535	-3.4676	0.00070
CAG-510	GA-GL	0.29496	0.383422	0.769283	0.44302
	GC-GL	-0.99876	0.432541	-2.30904	0.02240
Mediterraneibacter_A_155507	GA-GL	0.005361	0.332408	0.016127	0.98716
	GC-GL	-0.71899	0.374992	-1.91734	0.05723
Ruminococcus_B	GA-GL	-0.4037	0.318782	-1.26638	0.20748
	GC-GL	-1.07285	0.359621	-2.98327	0.00337
UBA3282	GA-GL	0.244606	0.348842	0.701195	0.48435
	GC-GL	-1.20134	0.393531	-3.05272	0.00271

Supplementary References

1. Bolyen E, Rideout JR, Dillon MR, Bokulich NA, Abnet CC, Al-Ghalith GA, et al. Reproducible, interactive, scalable and extensible microbiome data science using QIIME 2. Nat Biotechnol. 2019;37:852-7.
2. Callahan BJ, McMurdie PJ, Rosen MJ, Han AW, Johnson AJ, Holmes SP. DADA2: High-resolution sample inference from Illumina amplicon data. Nat Methods. 2016;13:581-3.
3. Quast C, Pruesse E, Yilmaz P, Gerken J, Schweer T, Yarza P, et al. The SILVA ribosomal RNA gene database project: improved data processing and web-based tools. Nucleic Acids Res. 2013;41:D590-6.
4. McMurdie PJ, Holmes S. phyloseq: an R package for reproducible interactive analysis and graphics of microbiome census data. PloS one. 2013;8:e61217.
5. Segata N, Izard J, Waldron L, Gevers D, Miropolsky L, Garrett WS, et al. Metagenomic biomarker discovery and explanation. Genome biology. 2011;12:R60.
6. Caspi R, Billington R, Keseler IM, Kothari A, Krummenacker M, Midford PE, et al. The MetaCyc database of metabolic pathways and enzymes - a 2019 update. Nucleic Acids Research. 2019;48:D445-D53.
7. Douglas GM, Maffei VJ, Zaneveld JR, Yurgel SN, Brown JR, Taylor CM, et al. PICRUSt2 for prediction of metagenome functions. Nature Biotechnology. 2020;38:685-8.
